# Understanding and Predicting Cognitive Improvement of Young Adults in Ischemic Stroke Rehabilitation Therapy

**DOI:** 10.3389/fneur.2022.886477

**Published:** 2022-07-13

**Authors:** Helard Becerra Martinez, Katryna Cisek, Alejandro García-Rudolph, John D. Kelleher, Andrew Hines

**Affiliations:** ^1^School of Computer Science, University of College Dublin, Dublin, Ireland; ^2^Information, Communication and Entertainment Research Institute, Technological University Dublin, Dublin, Ireland; ^3^Institut Guttmann Hospital de Neurorehabilitacio, Badalona, Spain; ^4^Universitat Autónoma de Barcelona, Cerdanyola del Vallés, Spain; ^5^Fundació Institut d'Investigació en Ciéncies de la Salut Germans Trias i Pujol, Badalona, Spain

**Keywords:** cognitive improvement, AI explainability, machine learning (ML), ischemic stroke, predictive models, cognitive therapy, web-based therapy

## Abstract

Accurate early predictions of a patient's likely cognitive improvement as a result of a stroke rehabilitation programme can assist clinicians in assembling more effective therapeutic programs. In addition, sufficient levels of explainability, which can justify these predictions, are a crucial requirement, as reported by clinicians. This article presents a machine learning (ML) prediction model targeting cognitive improvement after therapy for stroke surviving patients. The prediction model relies on electronic health records from 201 ischemic stroke surviving patients containing demographic information, cognitive assessments at admission from 24 different standardized neuropsychology tests (e.g., TMT, WAIS-III, Stroop, RAVLT, etc.), and therapy information collected during rehabilitation (72,002 entries collected between March 2007 and September 2019). The study population covered young-adult patients with a mean age of 49.51 years and only 4.47% above 65 years of age at the stroke event (no age filter applied). Twenty different classification algorithms (from Python's *Scikit-learn* library) are trained and evaluated, varying their hyper-parameters and the number of features received as input. Best-performing models reported Recall scores around 0.7 and F1 scores of 0.6, showing the model's ability to identify patients with poor cognitive improvement. The study includes a detailed feature importance report that helps interpret the model's inner decision workings and exposes the most influential factors in the cognitive improvement prediction. The study showed that certain therapy variables (e.g., the proportion of memory and orientation executed tasks) had an important influence on the final prediction of the cognitive improvement of patients at individual and population levels. This type of evidence can serve clinicians in adjusting the therapeutic settings (e.g., type and load of therapy activities) and selecting the one that maximizes cognitive improvement.

## 1. Introduction

A stroke event occurs when the blood supply to part of the brain is interrupted. There are two types of stroke depending on the problem in the blood vessel supplying the brain: ischemic stroke (blockage blood vessel) and hemorrhagic stroke (bleeding blood vessel). Out of these two, ischemic stroke is reported for almost 87% of all stroke episodes (1), with an increased incidence among adults aged 55 years and under in both the United States and Europe (2, 3). Among developed countries, stroke is considered the leading cause of death with a mortality rate of nearly 30% (4) and a disability rate of surviving patients close to 40% (5). For stroke survivors, the event can result in motor and/or cognitive impairments (e.g., body weakness, disturbances of cognitive functioning), and their outcome can vary from permanent disability to complete recovery (6, 7).

In this context, cognitive rehabilitation aims to maximize patients' recovery and achieve an optimal level of cognitive functioning to promote their reintegration into normal activities of daily living (8, 9). The clinical pathway of cognitive rehabilitation starts often with a clinical interview and several assessments to diagnose the patients' cognitive condition. This information is then used to formulate a personalized rehabilitation plan for the patient. Performance information can be recorded during the patient's therapy so the initial treatment plan can be adjusted. Although the rising of computerized cognitive programs has shown good advantages in therapy management and systematization (10, 11), a large part of the rehabilitation process still relies on the clinician's experience and availability. Thus, planning the best rehabilitation scheme for a large number of patients and closely monitoring their progress can turn into a very demanding task for a clinician (12, 13).

Due to their capacity to find patterns and relationships across data, machine learning (ML) models have shown promising results in solving complex problems in different fields, including medical diagnosis and prognosis prediction (14). In recent years, researchers have explored their integration into the stroke rehabilitation process, either as prediction tools or as part of larger clinical decision support systems (CDSS) (15–17). These ML-based tools can provide treatment recommendations and predictions of relevant outcomes such as the length of rehabilitation, the patient's performance throughout therapy, and the patient's cognitive and physical improvement after rehabilitation (18). For the specific case of cognitive improvement, having accurate predictions early in rehabilitation can help clinicians assemble a realistic therapy plan, adjust it to obtain better results, or anticipate additional care after the therapy.

Several studies have applied ML models to predict the cognitive condition of stroke survivors after therapy. These models often target scores from standardized scales such as the cognitive portion of the Functional Independence Measure (c-FIM), the Glasgow Outcome Scale (GOS), the National Institutes of Health Stroke Scale (NIHSS), or the Disability Rating Scale (DRS) (19–22). Other studies have developed models to predict outcomes for specific cognitive domains such as memory (risk of dementia) and language (aphasia recovery) (23, 24). Most of them rely on demographic and admission assessment variables to make predictions of a binary outcome (e.g., whether or not a patient achieves a specified score for a particular standardized scale), but only a few have explored the inclusion of therapy variables (22). Having reliable evidence of the impact that a therapy configuration (e.g., type and load of therapy activities) exerts on the cognitive rehabilitation outcome can help clinicians plan more effective rehabilitation programs. Moreover, it's been observed that despite the number of models developed over the years, clinical adoption has been cautious because of the limited capacity of models to explain their operations or outcomes and the ethical concerns this introduces regarding patient safety (25, 26). In this context, developing ML models with the capacity to produce actionable predictions and a sufficient level of explainability is crucial for clinical adoption, as pointed out by Stinear et al., in their review study (27).

This article presents a ML prediction model targeting the cognitive improvement after therapy of surviving patients with stroke. The model relies on patients' demographic characteristics, cognitive assessments at admission, and their corresponding therapy records. These records are integrated and pre-processed using standardized techniques commonly applied over data science projects and following clinicians' recommendations to avoid biased entries. Different models are trained and evaluated, varying the ML algorithm, their hyper-parameters and the number of features received as input. Then, predictions of the best-performing model are analyzed at global and individual levels using explanation methods. Through the development and analysis of this model this article seeks 1) to enable better design of rehabilitation therapy by exploring the impact of therapy configurations on patients' cognitive improvement; 2) to understand the underlying factors that improve/inhibit patients from improving on cognitive capacities after therapy using feature importance reports at individual and global levels; and 3) to explore visualization reports that could explain models' outcomes and bolster clinicians' trust.

The remainder of this document is divided as follows. Section 2 presents an overview of ML tools developed for stroke rehabilitation outcome prediction. In Section 3, the data and methods used in the study are described. Section 4 presents the data preparation, predictions, and model explanation results. Finally, Sections 5 and 6 present a detailed discussion and the overall conclusions of the study.

## 2. Related Study

Studies suggest that better rehabilitation outcomes can be achieved through 1) early rehabilitation and mobilization, 2) higher-intensity therapy, and 3) personalized therapy plans (28, 29). However, finding the best therapy options for an individual patient can be a very challenging task, even for a well-experienced clinician. Unlike traditional expert systems, which are restricted to hardcore knowledge and a set of rules, ML-based tools can learn solutions outside the expert knowledge and adapt themselves to each patient's condition providing more accurate and personalized solutions (30). In this context, ML models are an appealing option to assist clinicians during the rehabilitation process. The potential benefits of applying these new tools have drawn enormous attention to this field in recent years (27, 31). Most of the literature focuses on precision and personalized medicine in the area of diagnosis rather than the prognosis of cognitive improvement; however, the following studies represent some recent advancements in research into this area.

The authors of the study by Sale et al. (19), assessed the predictive value of common inflammatory biomarkers and other blood biomarkers in predicting cognitive and motor improvement upon completion of rehabilitation treatment. A two-step procedure was implemented consisting of feature selection among the biochemical and hematological parameters based on mutual information criteria and the application of a Support Vector Machine (SVM) classifier. This study confirmed previous reports of the prognostic value of biomarkers on motor function and cognitive performance in patients with post-stroke; however, it did not establish a link between the biomarkers and the type, intensity, and duration of the rehabilitation program and the corresponding impact of the rehabilitation on cognitive improvement.

The automatic cognitive prognosis model by Serrà et al. (32), implemented multiple methodologies, including decision tree learning, instance-based learning, probabilistic learning, and support vector machines, in order to build classifiers for cognitive improvement in the areas of attention, memory, and executive functioning. This study identified predictors such as patient age at the time of injury, the etiology, and pre-treatment neuropsychological evaluation scores as essential for accurate cognitive prognosis. However, this cross-sectional study focused on pre-treatment diagnosis data, without including cognitive rehabilitation treatment, discharge, or compliance data that would aid therapists in creating personalized rehabilitation programs.

In their research into the personalization of rehabilitation programs, García-Rudolph et al., built various classifiers based upon Knowledge Discovery in Databases (KDD) framework (33), focusing on pre-processing, patterns, and knowledge extraction. They implemented rehabilitation programs (cognitive computerized tasks) as sequences of sessions with pattern recognition utilizing methodologies such as association rules, classification, clustering and shallow neural models in order to assess patient rehabilitation task execution patterns with positive and negative responses to the rehabilitation treatment (34). This framework allowed for a finer representation of the design and configuration of successful rehabilitation programs for patients and the identification of small variations within these configurations that can aid in personalizing rehabilitation programs.

Finally, in the study by García-Rudolph (22), the authors evaluated and compared multiple predictive techniques and models to gain insight into the efficiency of rehabilitation programs in terms of the largest gain in function with the lowest duration of treatment. Traditional outcome prognosis models with demographic and clinical variables were compared to models augmented with additional variables describing the execution and configuration of cognitive computerized tasks, predicting optimal use of rehabilitation resources. The comparison and evaluation procedures involved robust parameter tuning, varied resampling methods of the traditional variables, with and without rehabilitation configuration variables, as well as model-dependent and model-independent ranking techniques to assess variables' importance. This study highlights the importance of utilizing variables describing the cognitive rehabilitation configuration. It provides therapists and clinicians with actionable predictions facilitating their decision-making for the rehabilitation intensity and duration and more specific interventions for patients.

## 3. Materials and Methods

**Figure 1:**
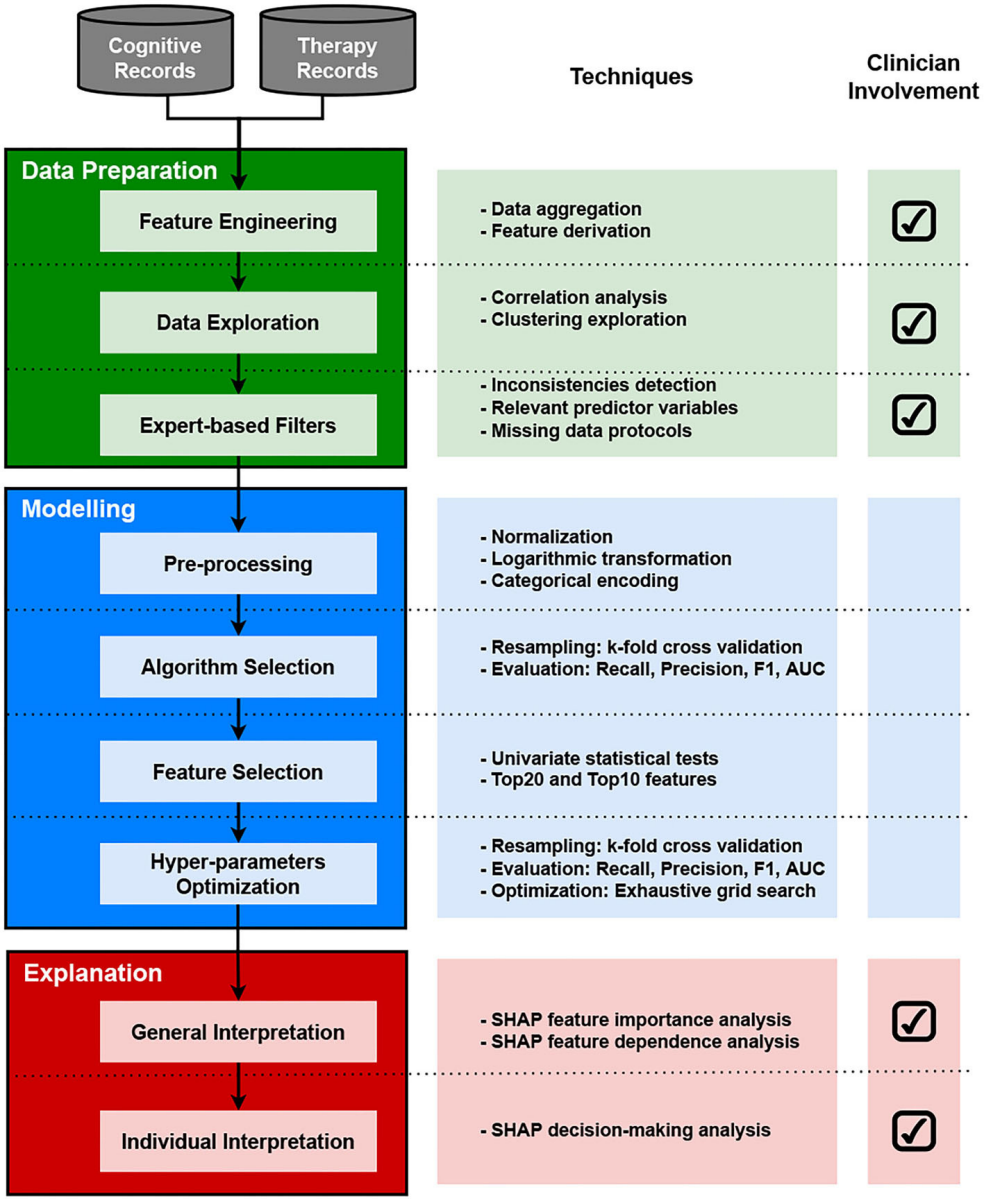
Diagram of the methodology used in this study.

The methodology followed in this study is presented in the workflow diagram in Figure 1. The diagram depicts three main phases with their associated tasks: data preparation, modeling, and explanation. It also includes a field indicating the involvement of healthcare professionals across tasks at each phase. This section presents a brief description of the data and methods used in this study.

### 3.1. Data Sources

This study relies on clinical data from ischemic stroke surviving patients admitted at the Acquired Brain Injury rehabilitation Department of Institut Guttmann (Barcelona, Spain). All participants were anonymized and non-identifiable. For this study, no specific written consent was required from participants; however, when admitted to Institut Guttmann's rehabilitation center, all patients provided written informed consent to be included in research studies carried out by the Institut Guttmann. The authors confirm that this study is compliant with the Helsinki Declaration of 1975, as revised in 2008, and it was approved by the Ethics Committee of Clinical Research of Guttmann Institut.

#### 3.1.1. Cognitive Records

Patients that are admitted at the Institut Guttmann rehabilitation center go through an interview and a set of neuropsychological assessments where demographic and clinical characteristics are entered into electronic health records from the hospital. A qualified neuropsychologist is in charge of conducting all neuropsychological assessments during admission. These standardized assessments aim to identify the level of cognitive impairment of the patient within different domains. Similarly, whenever a patient is to be discharged from the rehabilitation center (i.e., the patient completed the therapy at the center), the same set of assessments are conducted and recorded to analyze the patient's improvement. Table 1 lists the cognitive assessments included in this study, the cognitive domain they target, and the scale they use to report their results. Cognitive assessments report their results on different scales, as observed in the *Scale* column from Table 1. Except for TMT-A, TMT-B, and WCST Errors, cognitive assessments use scales where higher numbers are considered good outcomes.

**Table 1:** List of neuropsychological assessments administrated at the Guttmann Institut at admission and discharge.

**Assessment**	**Cognitive domain**	**Scale**
TB Personal Orientation (35)	Orientation	[0–7]
TB Spatial Orientation (35)	Orientation	[0–5]
TB Temporal Orientation (35)	Orientation	[0–23]
Digits Span (36)	Attention	[0–9]
TMT-A (37)	Attention	[0–Inf]
Stroop-Words (38)	Attention	[0–Inf]
Stroop-Color (38)	Attention	[0–Inf]
Stroop-Words/Colors (38)	Attention	[0–Inf]
TB Language Repetition (35)	Language	[0–10]
TB Language Denomination (35)	Language	[0–14]
TB Language Comprehension (35)	Language	[0–16]
Digit Span Backwards WAIS-III (36)	Memory	[0–8]
Numbers and Letters WAIS-III (36)	Memory	[0–16]
RAVLT Learning (39)	Memory	[0–75]
RAVLT Free Recall (39)	Memory	[0–15]
RAVLT Recognition (39)	Memory	[0–15]
TMT-B (37)	Executive Functions	[0–Inf]
WCST Categories (40)	Executive Functions	[0–6]
WCST Errors (40)	Executive Functions	[0–Inf]
Stroop-Interference (38)	Executive Functions	[Inf]
PMR (41)	Executive Functions	[0–Inf]
Visuospatial WAIS-III (36)	Visual	[0–Inf]
Images WAIS-III (36)	Visual	[0–20]
Cubes WAIS-III (36)	Visual	[0–Inf]
(*) NIHSS (42)	Overall impairment	[0–42]

TB, Test Barcelona; TMT, Trail Making Test; WAIS-III, Wechsler Adult Intelligence Scale 3rd version; RAVLT, Rey Auditory Verbal Learning Test; WCST, Wisconsin Card Sorting; NIHSS, National Institutes of Health Stroke Scale; (*), administrated only at admission.

#### 3.1.2. Therapy Records

The implementation of computerized rehabilitation platforms has gained increasing interest in recent years (10, 11). Studies have shown that these platforms can bring advantages such as therapy monitoring and feedback for patients, easy access (e.g., home-based care settings), and cost benefits (10). For therapy purposes, Institut Gutmann uses the Guttmann, NeuroPersonalTrainer^®^ (GNPT) (43) which is a rehabilitation platform for treatment systematization. The system contains 149 different cognitive rehabilitation web-based tasks assigned to the patient without a specific order. Therapy sessions are scheduled 2–5 times per week during the entire therapy, lasting from 2 to 6 months depending on the therapist's recommendation. A single therapy session can take 45 min to 1 h, and it can include 4–10 cognitive rehabilitation tasks. Each task targets a particular cognitive domain like orientation, attention, memory, language, executive functioning, calculus, gnosias, and praxias. After each task execution, the patient receives performance feedback ranging from 0 to 100 (0 being the lowest and 100 the highest). The system organizes this information as temporal entries and stores each task execution and its result with a set of relevant variables such as the date of execution and identifiers for the patient, the therapist, and the executed task. Despite this type of automation, therapists always have the capacity to select and adjust the treatment whenever they consider it necessary.

### 3.2. Study Population

This study included an initial cohort of 1,162 ischemic stroke patients admitted to the rehabilitation unit of the Institut Guttmann (Barcelona, Spain), between March 2007 and September 2019. For this study, the inclusion criteria, applied over the initial cohort, were based on the neuropsychologist's input: 1) admission at the rehabilitation center during the first 6 months since the stroke event; 2) patients who went through therapy and had records in the GNPT platform, and 3) a maximum length of therapy of 180 days. Applying these criteria resulted in the removal of 678 patients from the initial cohort leaving 484 patients with 77 descriptive variables.

Clinicians recommended another criterion to avoid including misleading entries. They commented that, in some cases, patients who could not complete their cognitive assessments in the first trial had to be assessed again on the same day. This procedure would erroneously record a therapy duration of 0 days. They recommended adding a minimum length of therapy (e.g., 14 days) to avoid including registries with inconsistent therapy lengths. The minimum length of therapy was established at 14 days, resulting in 475 patients with 77 descriptive variables.

Finally, both admission and discharge assessments were required to measure the improvement of a patient; however, as it is common when dealing with real-world data, the level of completeness was different for most cognitive variables. This posed a huge limitation because, in order to have a set of patients with all cognitive assessments, the dataset needed to be shrunk to the lowest completeness percentage of cognitive variables (around 26% of the current 475 patient entries). Removing all entries with missing assessments would have reduced the dataset to 81 patient entries and 77 accompanying variables. Although data imputation is usually applied to treat missing values, this practice is not always recommended when dealing with clinical data (44, 45). Thus, to mitigate the impact of removing entries with missing values, the assessments with the lowest completeness levels were selected for removal (assessments with less than 30% of completeness rate). Eight variables were selected for removal: Stroop-words, Stroop-color, Stroop-words/color, TMT-B, WCST categories, WCST errors, Stroop-interference, and Visuospatial WAIS-III. This procedure resulted in a dataset containing 201 patient entries with 69 descriptive variables. Although the number of entries is reduced, the approach aims to retain the data description capacity across all cognitive domains.

### 3.3. Feature Engineering

Through data aggregation, therapy variables like *daily sessions* and the *number of tasks* were constructed by summing the number of days and tasks registered in the GNPT therapy records. *Non executed tasks* and *non executed proportion* are two variables that capture the number of tasks with performance scores of “0.” Obtaining a score of “0” after a task execution indicates that the patient could not fulfill the minimum required by the rehabilitation task, which can be considered normal during an early stage of the rehabilitation process. However, from a neuropsychologist's perspective, patients that accumulate a large amount of “0” scores evidence difficulties completing their rehabilitation therapy. Therefore, variables that can capture this condition, such as *non executed tasks* and *non executed proportion*, might carry relevant information for cognitive outcome and therapy compliance predictions.

*Admission compliance* and *discharge compliance* are two variables derived from a neuropsychologist's expertise. From their analysis, it was observed that the level of completeness of cognitive assessments varied from admission to discharge. Neuropsychologists perceive this as a common occurrence since some patients tend to skip some assessments at admission because of their initial condition, especially when they are admitted very close to the date of the stroke event. Once they have completed their therapy and regained some of their cognitive capacities, they usually complete more assessments. On that account, the level of assessment compliance, i.e., the number of cognitive assessments patients complete at admission, can be a relevant indicator of the patient's cognitive capacity.

The *global improvement* was calculated by evaluating the difference between discharge and admission assessment scores and the summation of assigned markers. For instance, if the score difference between discharge and admission is positive, then the improvement of that assessment is “1,” if it is negative, then improvement is “–1,” and if there is no difference, the improvement is “0.” The *global improvement* is obtained by summing the improvement markers from all cognitive assessments and dividing the result by the number of assessments. The same process is carried out to generate improvement values for all cognitive domains (e.g., attention improvement, memory improvement, etc.). For cognitive assessments like TMT-A, TMT-B, and WCST Errors, which have an inverse score scale, a reverse procedure was applied to calculate the improvement.

Finally, *gain proportion* is introduced as a variable that tries to capture the relative gain after the execution of each task. This variable was derived from the weighted summation of all task results based on their performance ranges. For this study, different markers are assigned to each execution: a “0” marker is assigned to tasks with results of “0,” a “0.5” marker is assigned to tasks with results falling in “1–64” and “86–100” ranges, finally, a marker “1” is assigned to tasks with results in the range of “65–85.” The assignment of these markers is based on the assumption that “65–85” is the optimal range of performance for task execution (46). The intuition behind the definition of this optimal therapeutic range is that it is best to avoid results that are too low (“1–64”) from tasks that are too difficult to get responses from a damaged area of the brain; or extremely high results (“86–100”) from tasks that are too simple to exercise such damaged areas. During their therapy, a patient accumulates different markers for each task execution. These markers are then summed to obtain the total gain after therapy. The *gain proportion* results from dividing the *total gain* by the *number of tasks*. Equivalent variables are calculated for each cognitive domain (*attention execution gain, memory execution gain*, etc.).

### 3.4. Data Exploration

In addition to measures like mean, variance, minimum, and maximum, which can help describe the variables individually, correlation and clustering analyses are applied over the rehabilitation dataset to explore the relevant relationships among the available features.

For this study, correlation heatmaps were used to understand how the numeric variables relate to each other within the rehabilitation dataset. This type of analysis is a good starting point to identify multicollinearity scenarios where two or more variables present a strong correlation. From a data scientist's standpoint, evidence from this analysis can serve to reduce overlap in input variables by removing variables that do not add prediction power to the model or to assist in mitigating sparse data by complementing missing information from variables. For the cluster exploration, the analysis was carried out using the Principal Component Analysis (PCA) and the t-Distributed Stochastic Neighbourhood Embedding (t-SNE) techniques (47, 48). The objective was to identify possible patterns, unseen structures, or possible anomalies across the available demographic, cognitive, and therapy data. Both PCA and t-SNE are dimensionality reduction techniques that ease data visualization and allow the discovery of veiled insights from it. They differ in the sense that PCA is linear and deterministic, and it tries to retain the global structure of the data; meanwhile, t-SNE is non-linear and non-deterministic (randomized), and it tries to preserve the local structure of data.

### 3.5. Modeling

#### 3.5.1. Cognitive Outcome

For this study, the *global improvement* was considered as the target variable to predict the cognitive outcome. The *global improvement* combines improvement indicators of all standardized cognitive assessments administered to the patient, which can help describe the patient's overall status after therapy. This variable can rely on demographic, cognitive, and therapy variables to act as predictor features. For this study, the *global improvement* was adapted to fit a common binary classification problem. A threshold was used to separate patients with positive improvement scores and negative (or zero) improvement scores. (Class “0”: *global improvement <=0*, Class “1”: *global improvement>0*). From a binary classification perspective, the positive class is referred to as the class of interest for a defined problem. In this study, the main focus was to identify patients “at risk,” that is, patients reporting negative or zero *global improvement* values. Therefore, Class “0” was selected as the positive class.

#### 3.5.2. Pre-processing

A pre-processing step is recommended to standardize and format the features before training the ML model. Although some families of ML algorithms are able to handle features with different scales and formats, pre-processing before training the model is recommended to guarantee better performance (49). It is also desired for features to maintain a normal distribution so they can be applied to different ML algorithms. For this study, input variables were scaled using the *StandardScaler* method from the *Scikit-learn Python* library. This method removes the mean of features (i.e., set it to zero) and scale values to unit variance. For variables with a skewed distribution, the *Scikit-learn* implementation of the *PowerTransformer* method (50) was used to treat features to obtain a more “Gaussian-like” distribution. Finally, nominal features were encoded and transformed into numeric features to allow for performing mathematical operations.

#### 3.5.3. Classification Algorithms

Twenty different classification algorithms from nine different families were considered for this study: five Linear Models, one Nearest Neighbor, two Decision Trees, two Support Vector Machines, two Naive Bayes, five Ensemble Methods, one Gaussian Process, one Linear Discriminant Analysis, and one Boosted Trees. The implementation of these algorithms, available at the *scikit-learn* library and the *xgboost* package for *Python*, were used for training and evaluation.

#### 3.5.4. Performance Evaluation

The algorithms' performance was measured using standard classification metrics like F1 score, Recall, Precision, and the Area Under the Receiver Operator Characteristic Curve (ROC-AUC). The Recall score was selected as the main evaluation criteria as it prioritizes the correct positive instances that are correctly classified, i.e., patients with zero or negative improvement scores (class “0”). To cope with a low number of entries in the dataset, a k-fold (*k* = 5) cross-validation re-sampling method with 5 repetitions was used to evaluate the performance of different classification algorithms. For this task, the algorithms were assessed using default *Scikit-learn* settings with no customized parameters.

#### 3.5.5. Feature Selection

Before the optimization, feature selection was carried out to identify the most important features using the model performance as the main criteria. Different from the selection made using the clinicians and data scientists as a reference, this procedure uses the relationship between input and target variables to rank the contribution of each input feature. The idea is to verify the models' performance using different amounts of input features. Using fewer features while maintaining good performance is a desirable characteristic as it reduces the number of assessments required by the patient (51). This also aligns with the data minimization principle from the European General Data Protection Regulations (GDPR) (52). The feature selection step was carried out using the *SelectKBest* method implemented in *Scikit-learn*. For a classification problem, this method uses an implementation of the ANOVA f-test to rank features and select the ones with higher scores. For this case study, the entire set of features, plus the best 20 and 10 features, were selected and included in the hyper-parameter optimization setting.

#### 3.5.6. Hyper-Parameters Optimization

To find the best performing parameters, a grid-search was carried out using the *GridSearchCV* method implemented in *Scikit-learn* along with a k-fold (*k* = 5) cross-validation method. Table 2 presents the sets of values selected to carry out the grid-search for each parameter and pre-selected algorithm. For the *ExtraTreesClassifier* and *RandomForestClassifier*, similar parameters were set for optimization; meanwhile, the *KNeighborsClassifier, XGBClassifier*, and *LogisticRegression* had specific tuning parameters. The models were optimized based on their Recall performance. Then, using the best parameters from the Recall optimization, F1, Precision, and ROC-AUC scores were evaluated *via* k-fold (*k* = 5) cross-validation.

**Table 2:** Sets of hyper-parameters used for the grid-search corresponding to the pre-selected classification algorithms.

**Algorithm**	**Hyper-paramets set**
ExtraTreesClassifier	n_estimators = [10, 100, 1,000]
	max_depth = [3, 7, 9]
	min_samples_split=[2,10,20]
	criterion=[“gini,” “entropy”]
	min_weight_fraction_leaf = [0,0.2,0.3,0.5]
RandomForestClassifier	criterion=[“gini,” “entropy”]
	n_estimators = [10, 100, 1,000]
	max_features = [“sqrt,” “log2”]
	max_depth = [9, 15]
	min_samples_split=[2,10,20]
	min_weight_fraction_leaf = [0,0.2,0.5]
KNeighborsClassifier	n_neighbors = [2, 10, 21]
	weights = [“uniform,” “distance”]
	metric = [“euclidean,” “manhattan,” “minkowski”]
XGBClassifier	eta = [0.001, 0.01, 0.1, 0.2, 0.3]
	gamma = [0.05, 0.5, 1, 1.5]
	min_child_weight = [5, 7, 9, 10]
	subsample = [0.5, 0.8, 1]
	colsample_bytree = [0.6, 0.8, 1]
	lambda_par = [0.1, 0.5, 1]
LogisticRegression	solver = [“newton-cg,” “lbfgs,” “liblinear”]
	penalty = [“L2”]
	C = [100, 10, 1.0, 0.1, 0.01]
	max_iter = [1,000]

### 3.6. Explanation Methods

In addition to the classification metrics that report the performance of the model, explanatory material that allows clinicians to interpret the inner workings of the model is needed (53–55). From several explanation classes, clinicians identified that having a report of the most influential features on a model's outcome was critical, as it allowed them to compare the model's decision process with their clinical judgment process (53). The SHapley Additive exPlanations (SHAP) method (56) was used to analyze the trained models and describe the model's inner rationale. SHAP is a unified framework based on six feature importance methods that facilitate the interpretation of ML models' predictions. The underlying logic behind SHAP's inner workings is based on evaluating the trained model using different sets of feature permutations and calculating each feature's average contribution to the model's output; this average value is called the SHAP value. Computing SHAP values for each input feature allows interpreting each feature's average contribution to the model's prediction. For this study, the implementation of the SHAP library for *Python* was used to examine the trained models.

## 4. Results

### 4.1. Experiment Data

Table 3 presents basic statistics of all demographic, cognitive, and therapy variables from the rehabilitation dataset to help understand the characteristics of the available data. The TMT-A and TMT-B assessments showed higher standard deviation values (48.43 and 82.51); meanwhile, TB Personal and TB Language Repetition assessments reported the lowest variability among assessments (0.00). For therapy variables, the total number of tasks showed a SD value of 85.22, which evidence the load of therapy differences among patients. The same variable reported a maximum value of 480, meaning that a single patient executed this amount of rehabilitation tasks. Table 4 presents the task performance (grouped by ranges) given by the GNPT system after each task execution. Executed tasks with a performance result of “0” represent 16.2% of all executed tasks. Results falling in the “65–85” range represent 18.9% of the executed tasks. Within the GNPT platform, this range is considered optimal since it balances the cognitive gain of the patient (46). The distribution of executed tasks shows that memory tasks are the most repeated, as they represent 40.2% of all performed tasks. Meanwhile, attention and executive functions cover 19.5% and 29.4% of all entries.

**Table 3:** Basic statistics of demographic, cognitive and therapy variables from the rehabilitation dataset.

**Group**	**Variable**	**Mean**		**SD**		**Min**		**Max**	
Demographic									
	Age	49.70		10.18		16.74		81.92	
	Age at injury (mc)	49.52		10.18		16.65		81.84	
	Time since injury in days	66.46		40.30		1.60		173.43	
	Length of therapy	65.35		30.79		15.00		173.00	
	Sex (c)								
	Male	N/A		N/A		N/A		N/A	
	Female	N/A		N/A		N/A		N/A	
	Marital status (c)								
	Married	N/A		N/A		N/A		N/A	
	Single	N/A		N/A		N/A		N/A	
	Divorce	N/A		N/A		N/A		N/A	
	Separate	N/A		N/A		N/A		N/A	
	Widow	N/A		N/A		N/A		N/A	
Cognitive									
	Admission compliance	0.90		0.11		0.67		1.00	
	Discharge compliance (mc)	0.94		0.09		0.67		1.00	
	Global improvement (t)	0.18		0.21		-0.50		0.75	
	Attention improvement (mc)	0.02		0.17		-0.33		0.67	
	Orientation improvement (mc)	0.27		0.40		-0.80		1.00	
	Language improvement (mc)	0.04		0.15		-0.33		0.67	
	Memory improvement (mc)	0.27		0.44		-1.00		1.00	
	Ex. Functions improvement (mc)	0.23		0.39		-1.00		1.00	
	Visual improvement	0.31		0.41		-0.67		1.00	
	NIHSS	9.86		4.65		1.00		22.00	
	**Variable**	**Mean**		**SD**		**Min**		**Max**	
		**Adm**	**Dis**	**Adm**	**Dis**	**Adm**	**Dis**	**Adm**	**Dis**
Orientation	TB Personal Orientation	6.99	7.00	0.12	0.00	6	7	7	7
	TB Spatial Orientation	4.97	4.99	0.17	0.10	4	4	5	5
	TB Temporal Orientation	22.59	22.71	1.41	1.22	12	11	23	23
Attention	Digits Span	5.91	6.03	1.10	1.07	3	4	9	9
	TMT-A	67.44	53.42	48.43	31.70	4	6	289	240
	Stroop - Words (md)	78.43	82.05	16.37	15.51	37	40	123	125
	Stroop - Color (md)	55.51	57.70	12.45	12.54	23	27	89	90
	Stroop - Words/Colors (md)	32.04	34.03	10.71	11.04	6	8	85	73
Language	TB Language Repetition	9.99	10.00	0.10	0.00	9	10	10	10
	TB Language Denomination	13.96	14.00	0.27	0.00	11	14	14	14
	TB Language Comprehension	15.78	15.91	0.76	0.50	9	12	16	16
Memory	Digit Span Backwards WAIS-III	4.19	4.35	0.98	0.98	2	2	7	8
	Numbers and Letters WAIS-III	8.14	8.72	2.61	2.52	1	3	14	15
	RAVLT Learning	42.14	46.23	10.66	11.49	21	9	70	70
	RAVLT Free Recall	8.26	9.35	3.51	3.41	0	0	15	15
	RAVLT Recognition	11.44	12.19	3.91	3.20	0	1	15	15
Executive Functions	TMT-B (md)	141.13	112.07	82.51	48.63	30	30	565	300
	WCST Categories (md)	4.11	4.24	2.11	2.13	0	0	6	6
	WCST Errors (md)	18.36	16.25	15.07	14.64	0	0	63	72
	Stroop - Interference (md)	-0.21	0.41	7.20	7.48	-21	-22	35	25
	PMR	31.93	35.10	13.25	13.30	3	5	72	84
Visual	Visuospatial WAIS-III (md)	41.21	46.23	15.59	16.04	10	13	92	92
	Images WAIS-III	19.30	19.67	1.49	0.94	11	14	20	20
	Cubes WAIS-III	26.96	30.31	11.97	11.94	2	6	66	66
**Variable**		**Mean**		**SD**		**Min**		**Max**	
Therapy									
	Daily sessions	12.15		7.91		1.00		52.00	
	Total number of tasks (mc)	111.89		85.22		2.00		480.00	
	Total non executed tasks (mc)	15.84		18.93		0.00		117.00	
	Non executed proportion	0.14		0.10		0.00		0.53	
	Total gain proportion (mc)	0.53		0.08		0.25		0.75	
Attention	Number of tasks (mc)	20.93		18.36		1.00		123.00	
	Task proportion	0.19		0.10		0.01		0.67	
	Non executed tasks	2.01		3.85		0.00		40.00	
	Execution gain	11.25		10.22		0.00		72.00	
Memory	Number of tasks (mc)	45.65		40.40		2.00		294.00	
	Task proportion	0.40		0.15		0.07		1.00	
	Non executed tasks	5.22		8.08		0.00		53.00	
	Execution gain	25.57		23.53		0.50		181.00	
Ex. Functions	Number of tasks (mc)	38.16		30.58		1.00		170.00	
	Task proportion	0.35		0.13		0.05		0.75	
	Non executed tasks	8.12		9.13		0.00		62.00	
	Execution gain	18.08		15.17		0.00		96.50	
Language	Number of tasks (mc)	8.86		13.35		1.00		55.00	
	Task proportion	0.16		0.28		0.01		1.00	
	Non executed tasks	0.71		1.10		0.00		3.00	
	Execution gain	5.04		9.10		0.50		37.00	
Orientation	Number of tasks (mc)	4.31		5.27		1.00		31.00	
	Task proportion	0.03		0.04		0.00		0.20	
	Non executed tasks	0.39		1.30		0.00		10.00	
	Execution gain	2.06		2.58		0.00		15.00	
Calculus	Number of tasks (mc)	11.81		11.45		1.00		62.00	
	Task proportion	0.09		0.07		0.01		0.31	
	Non executed tasks	1.42		2.32		0.00		11.00	
	Execution gain	6.25		6.29		0.00		36.50	
Gnosias	Number of tasks (mc)	8.50		14.68		1.00		81.00	
	Task proportion	0.06		0.09		0.01		0.49	
	Non executed tasks	0.47		1.47		0.00		8.00	
	Execution gain	4.81		8.11		0.00		40.00	
Praxias	Number of tasks (mc)	3.71		2.99		2.00		12.00	
	Task proportion	0.02		0.01		0.01		0.07	
	Non executed tasks	0.24		0.64		0.00		2.00	
	Execution gain	1.82		1.74		0.00		6.50	

*N = 201; Adm, admission; Dis, discharge; c, categorical variable; mc, removed to prevent multicollinearity issues; md, removed to prevent missing data issues; t, target variable*.

**Table 4:** Summary report from temporal records of the GNPT platform.

**Variable**	**n = 72,002**
**Task performance by result ranges**, **n** **(%)**	
[0]	11,669 (16.2%)
[1–64]	23,501 (32.6%)
[65–85]	13,573 (18.9%)
[86–100]	23,259 (32.3%)
**Number of tasks per cognitive function**, **n** **(%)**	
Attention	14,015 (19.5%)
Memory	28,963 (40.2%)
Ex. Functions	21,172 (29.4%)
Language	1,923 (2.7%)
Orientation	979 (1.4%)
Calculus	2,377 (3.3%)
Gnosias	2,379 (3.3%)
Praxias	158 (0.2%)

### 4.2. Exploratory Analysis

**Figure 2:**
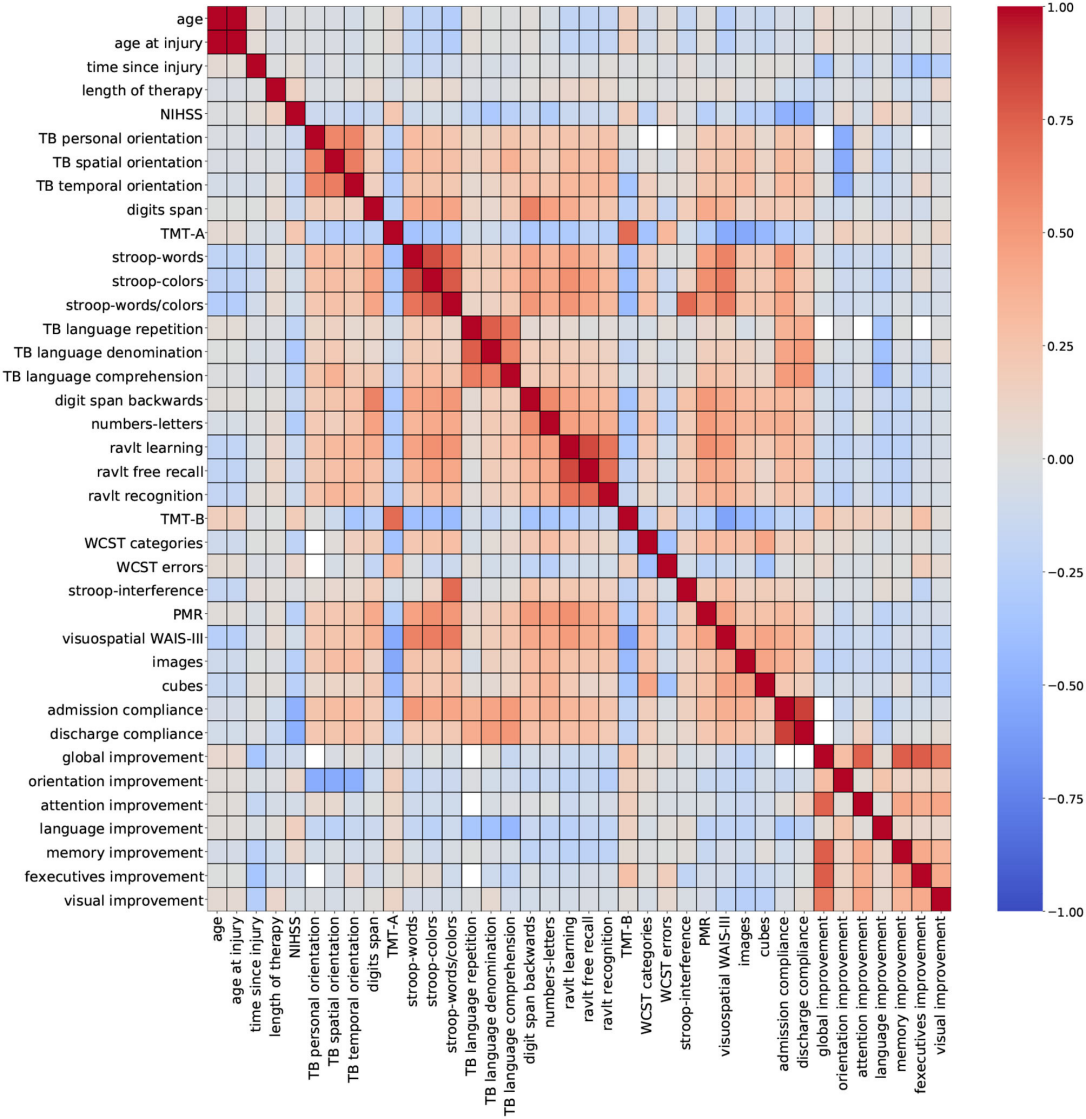
Correlation outcomes from demographic + cognitive variables. Red: strong positive correlation (+1), blue: strong negative correlation (–1).

**Figure 3:**
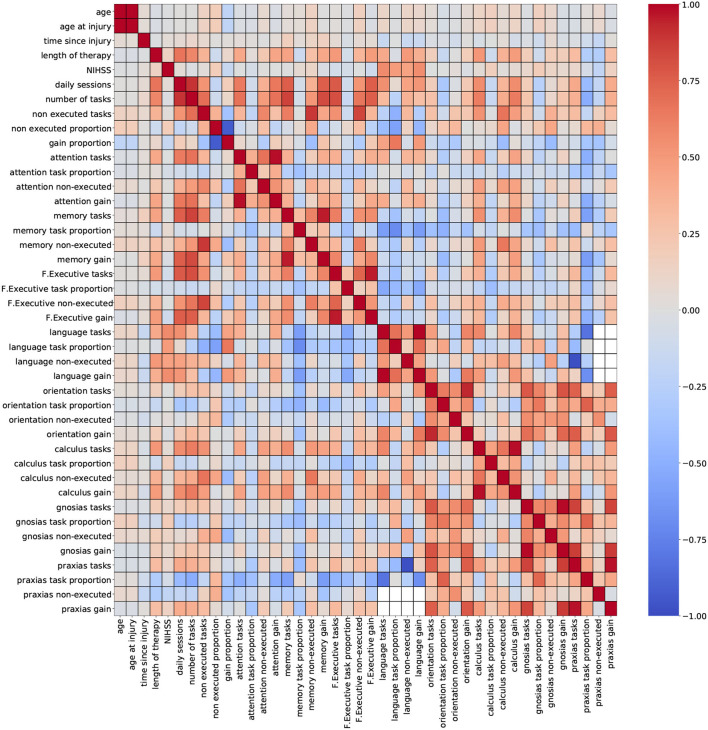
Correlation outcomes from demographic + therapy variables. Red: strong positive correlation (+1), blue: strong negative correlation (–1).

For this study, two correlation heatmaps were generated using the demographic, cognitive, and therapy variables. For visualization purposes, correlation outcomes are presented in separated plots: Figure 2 (demographic + cognitive) and Figure 3 (demographic + therapy). Color bars were used to a show strong positive correlation (red) and a strong negative correlation (blue).

Figure 2 (demographic + cognitive) shows several clusters of low-to-moderate positive correlation coefficients (0.2–0.6), corresponding to standardized cognitive assessment variables. Smaller clusters of strong correlation coefficients (0.7–0.8) belonging to variables from the same group of standardized assessments are observed near the diagonal. This trait was also observed for variables admission compliance and discharge compliance. These results show that some assessments carry very similar information, especially when they belong to the same cognitive domain. Other variables like age, time since injury, NIHSS, and global improvement reported low-to-moderate negative correlation coefficients (−0.2 to −0.6). For some variables, a strong correlation is expected, and it can be easily explained like for the pair of features age and age at injury. In a stroke rehabilitation context, it is expected that patients start their therapy within the first 6 months of a stroke occurring; thus, it is likely that these two variables have close values. On that account, including only one of these variables in the descriptor set might be sufficient for modeling purposes.

Figure 3 (demographic + therapy) shows a more sparse distribution of correlation outcomes. For this analysis, strong correlation clusters are more abundant, and they are related, in most cases, to three variables: daily sessions, number of tasks, and non executed tasks. The heatmap shows that these clusters are formed across each cognitive domain, and that they are stronger among the ones with a higher number of tasks executions (attention, memory and executive functions). The pair of variables daily sessions and number of tasks reported strong correlation coefficients. Since they both depend on the length of the therapy (more therapy sessions will result in more executed tasks), a strong correlation between these two variables is expected. Again, keeping only one of these variables for modeling purposes seems to be a valid suggestion.

Including groups of highly correlated variables as input to train an ML model might not affect the model's performance, but it can reduce the capacity to interpret the effect of variables on the model's outcome. Thus, to develop explainable ML tools, data scientists recommend handling multicollinearity instances before training the model (57). Based on the findings from the correlation analysis, a few variables were selected for removal to mitigate possible overlapping effects. Thirteen variables were removed from the dataset: age at injury, discharge compliance, number of tasks, non executed tasks, gain proportion, attention tasks, memory tasks, F. executive tasks, language tasks, orientation tasks, calculus tasks, gnosias tasks, and praxias tasks. After this procedure, the number of available variables is 56 (54 numeric and 2 nominal).

**Figure 4:**
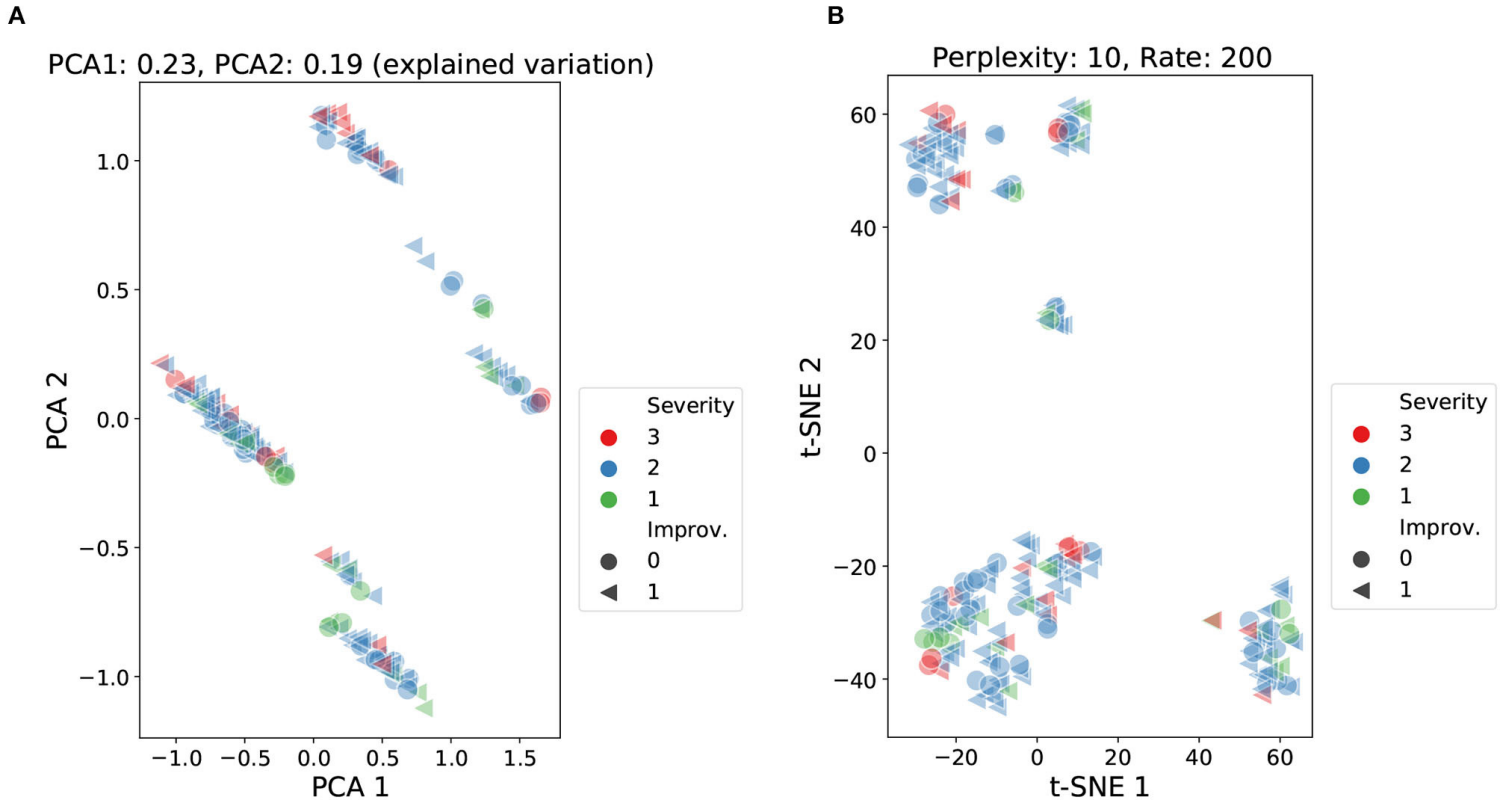
Cluster analysis using PCA and t-SNE over demographic, cognitive, and therapy variables. Data points labeled according to severity (NIHSS score) and improvement (global improvement). PCA: accumulated explained variance 0.42 **(A)**. t-SNE: perplexity 10, learning rate 200 **(B)**.

Following the exploratory stage, a cluster analysis was carried out using the Principal Component Analysis (PCA) and the t-Distributed Stochastic Neighbourhood Embedding (t-SNE) methods (47, 48). The objective was to identify possible patterns, unseen structures, or possible anomalies across the available demographic, cognitive, and therapy data. Figure 4 presents 2D visualizations generated using the PCA and t-SNE methods. Each data point, representing a patient, was labeled according to the severity of the condition of the patient (from the NIHSS score) and the improvement after the therapy (from the global improvement variable). This type of visualization can help display homogeneous groups of patients and verify if predictor variables correlate with the target labels of the model.

The PCA analysis presented in Figure 4A reported an accumulated explained variance of 0.42 for the two components (PCA1 and PCA2). Figure 4A shows four clusters plus two smaller sets of data points slightly separated from two of the main clusters. For the t-SNE method, different perplexity and rate parameters were tested at different runs of the method. The scatter plot in Figure 4B shows the results from setting the perplexity parameter at 10 and the learning rate at 200. The scatter plot reported three main clusters plus a few smaller sets of data points. Both methods were able to produce relatively dense clusters and separate classes at some level. Yet, the formation of these clusters does not seem to be linked to the level of stroke severity (NIHSS score) or the outcome of patients (global improvement), as no patterns are perceived among the shapes and colors of the data points. These findings highlight that both severity and improvement labels were not strongly correlated with the information carried by the input features.

### 4.3. Cognitive Improvement Prediction

**Figure 5:**
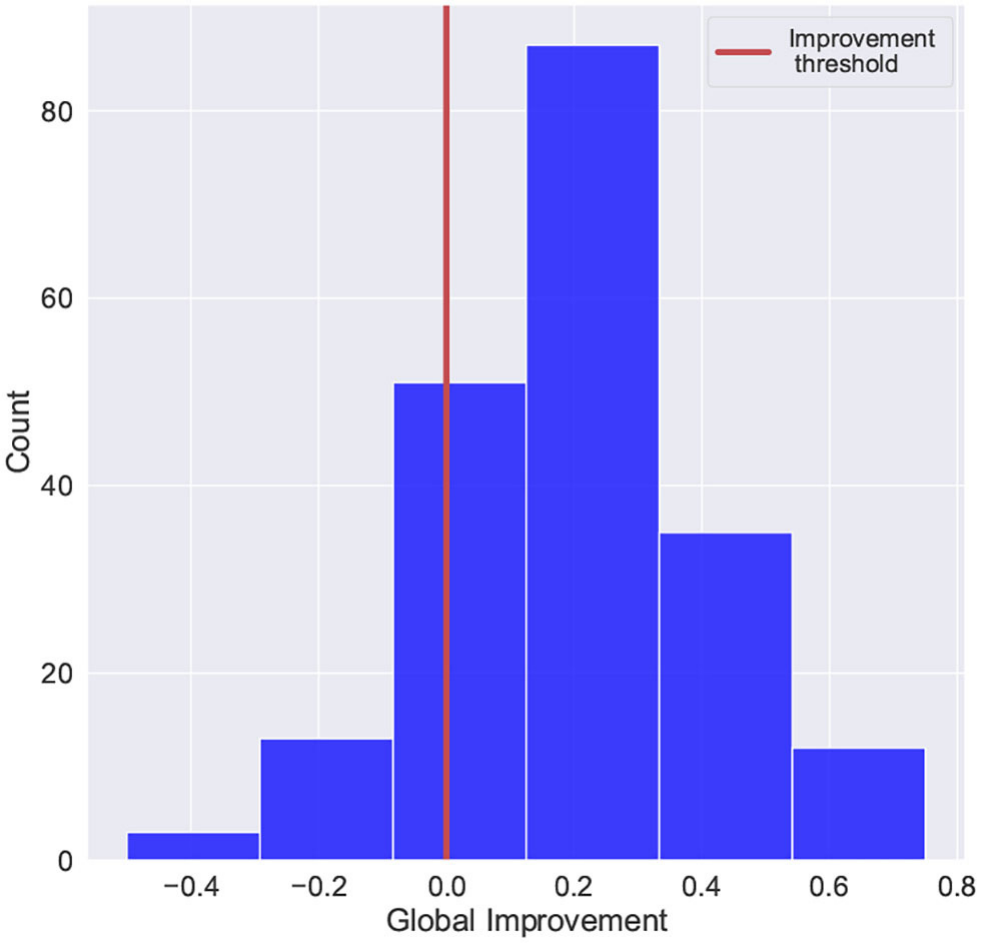
Scores distribution of global improvement. Class “0”: global improvement <=0, Class “1”: global improvement>0.

For this study, the global improvement was adapted to fit a common binary classification problem. Figure 5 shows the distribution of the improvement scores and the threshold used to separate patients with positive improvement scores and negative (or zero) improvement scores (Class “0”: global improvement <=0, Class “1”: global improvement>0). The histogram shows that there were fewer cases with negative or no improvement scores (58 out of 201 registries), which evidence the imbalanced distribution of the target.

Twenty different ML classification algorithms were trained and evaluated using a k-fold (k = 5) cross-validation re-sampling method with 5 repetitions. Table 5 list the ten best-performing algorithms and their corresponding results for Recall, F1, Precision, and AUC-ROC scores. The algorithms reported Recall scores around 0.70; meanwhile, the reported F1 scores reached 0.63. Based on these results, five algorithms were pre-selected to be considered for hyper-parameter optimization: RandomForestClassifier, ExtraTreesClassifier, KNeighborsClassifier, XGBClassifier, and LogisticRegression.

**Table 5:** Evaluation of the classification algorithms without hyper-parameter tuning.

**Algorithm**	**F1**	**Recall**	**Precision**	**AUC**
RandomForestClassifier	0.638 (0.08)	**0.701 (0.05)**	0.652 (0.08)	0.526 (0.05)
ExtraTreesClassifier	0.630 (0.08)	**0.697 (0.07)**	0.640 (0.11)	0.528 (0.05)
KNeighborsClassifier	0.614 (0.10)	**0.671 (0.08)**	0.606 (0.13)	0.512 (0.06)
XGBClassifier	0.636 (0.08)	**0.664 (0.07)**	0.642 (0.08)	0.543 (0.07)
LogisticRegression	0.643 (0.06)	**0.658 (0.06)**	0.646 (0.08)	0.549 (0.08)
RidgeClassifier	0.633 (0.05)	**0.651 (0.05)**	0.637 (0.07)	0.534 (0.07)
BaggingClassifier	0.610 (0.08)	**0.642 (0.06)**	0.632 (0.06)	0.533 (0.07)
LinearSVC	0.634 (0.06)	**0.641 (0.07)**	0.639 (0.07)	0.544 (0.07)
LinearDiscriminant Analysis	0.614 (0.06)	**0.625 (0.06)**	0.616 (0.07)	0.515 (0.07)
BernoulliNB	0.611 (0.06)	**0.619 (0.07)**	0.626 (0.07)	0.516 (0.07)

*Results sorted by the mean Recall scores accompanied by their corresponding SD. Re-sampling at k-fold (k = 5) cross-validation with 5 repetitions. The bold values indicated to highlight the recall columns*.

All five pre-selected algorithms were trained using three different sets of input features: “All”: no feature selection, “20”: best 20 features, and “10”: best 10 features. Table 6 presents the optimized values for each parameter for all five classification algorithms and the corresponding sets of input features; meanwhile, the performance results from the Recall, F1, Precision, and AUC-ROC scores are presented in Table 7. These results showed no significant improvement for any algorithm compared to the results presented in Table 5. Recall and F1 scores floated around 0.7 and 0.6, respectively, for most classification algorithms. In addition, no considerable performance differences were reported regarding the number of features used to train the models. These results imply that it is possible to train a model with fewer features at a low cost in performance. Despite the room for performance improvement, these results show the model's ability to identify patients with poor cognitive improvement after therapy.

**Table 6:** Best performing sets of hyper-parameters gathered during the grid-search optimization process.

**Algorithm**	**Hyper-parameters**
ExtraTreesClassifier_All	criterion: gini, max_depth: 9, min_samples_split: 2, min_weight_fraction_leaf: 0, n_estimators: 1,000
ExtraTreesClassifier_20	criterion: gini, max_depth: 3, min_samples_split: 2, min_weight_fraction_leaf: 0.2, n_estimators: 10
ExtraTreesClassifier_10	criterion: gini, max_depth: 3, min_samples_split: 2, min_weight_fraction_leaf: 0.2, n_estimators: 10
RandomForestClassifier_All	criterion: entropy, max_depth: 15, max_features: log2, min_samples_split: 10, min_weight_fraction_leaf: 0, n_estimators: 1,000
RandomForestClassifier_20	criterion: entropy, max_depth: 15, max_features: sqrt, min_samples_split: 20, min_weight_fraction_leaf: 0.2, n_estimators: 10
RandomForestClassifier_10	criterion: gini, max_depth: 15, max_features: log2, min_samples_split: 2, min_weight_fraction_leaf: 0.2, n_estimators: 10
KNeighborsClassifier_All	metric: euclidean, n_neighbors: 17, weights: distance
KNeighborsClassifier_20	metric: manhattan, n_neighbors: 19, weights: uniform
KNeighborsClassifier_10	metric: euclidean, n_neighbors: 19, weights: uniform
XGBClassifier_All	colsample_bytree: 0.6, eta: 0.01, gamma: 1, min_child_weight: 5, reg_lambda: 0.5, subsample: 0.8
XGBClassifier_20	colsample_bytree: 0.8, eta: 0.1, gamma: 0.5, min_child_weight: 9, reg_lambda: 0.1, subsample: 0.5
XGBClassifier_10	colsample_bytree: 0.6, eta: 0.001, gamma: 0.05, min_child_weight: 5, reg_lambda: 0.1, subsample: 0.5
LogisticRegression_All	C: 0.01, max_iter: 300, penalty: l2, solver: newton-cg
LogisticRegression_20	C: 0.01, max_iter: 300, penalty: l2, solver: newton-cg
LogisticRegression_10	C: 0.01, max_iter: 300, penalty: l2, solver: newton-cg

*Recall scores as evaluation criteria. Results reported for the different number of input features. “All”: no feature selection, “20”: best 20 features, “10”: best 10 features. Re-sampling at k-fold (k = 5) cross-validation with five repetitions*.

**Table 7:** Evaluation of the pre-selected algorithms with hyper-parameters tuning.

**Algorithm**	**F1**	**Recall**	**Precision**	**AUC**
ExtraTreesClassifier_All	0.637 (0.09)	**0.714 (0.06)**	0.640 (0.15)	0.536 (0.05)
ExtraTreesClassifier_20	0.593 (0.08)	**0.711 (0.06)**	0.510 (0.08)	0.500 (0.00)
ExtraTreesClassifier_10	0.593 (0.08)	**0.711 (0.06)**	0.510 (0.08)	0.500 (0.00)
RandomForestClassifier_All	0.612 (0.08)	**0.713 (0.06)**	0.610 (0.13)	0.512 (0.03)
RandomForestClassifier_20	0.597 (0.08)	**0.713 (0.06)**	0.515 (0.09)	0.502 (0.01)
RandomForestClassifier_10	0.593 (0.08)	**0.714 (0.06)**	0.508 (0.08)	0.500 (0.01)
KNeighborsClassifier_All	0.606 (0.07)	**0.714 (0.06)**	0.596 (0.13)	0.508 (0.02)
KNeighborsClassifier_20	0.624 (0.08)	**0.708 (0.06)**	0.654 (0.12)	0.522 (0.04)
KNeighborsClassifier_10	0.609 (0.09)	**0.692 (0.07)**	0.591 (0.13)	0.507 (0.04)
XGBClassifier_All	0.617 (0.09)	**0.713 (0.07)**	0.624 (0.16)	0.517 (0.04)
XGBClassifier_20	0.608 (0.10)	**0.716 (0.07)**	0.556 (0.15)	0.516 (0.04)
XGBClassifier_10	0.593 (0.08)	**0.712 (0.06)**	0.510 (0.08)	0.500 (0.00)
LogisticRegression_All	0.607 (0.07)	**0.708 (0.06)**	0.595 (0.13)	0.507 (0.03)
LogisticRegression_20	0.607 (0.07)	**0.709 (0.06)**	0.595 (0.13)	0.507 (0.03)
LogisticRegression_10	0.604 (0.08)	**0.712 (0.06)**	0.567 (0.13)	0.507 (0.02)

*Recall scores as evaluation criteria. Results reported for the different number of input features. “All”: no feature selection, “20”: best 20 features, “10”: best 10 features. Re-sampling at k-fold (k = 5) cross-validation with 5 repetitions The bold values indicated to highlight the recall columns*.

### 4.4. Explanation Reports

For this study, the feature importance analysis was organized at two levels: a general interpretation covering the model's behavior and feature relevance across the entire dataset and an individual interpretation exploring the cases of four particular subjects from the dataset. The analysis was made using the best performing hyper-parameters found during the optimization process (Table 6) and the entire set of features since no significant difference was observed among the input sets of features. Due to the absence of an outstanding algorithm in terms of performance, the XGBClassifier_All model was selected for the explanatory analysis.

#### 4.4.1. General Interpretation

**Figure 6:**
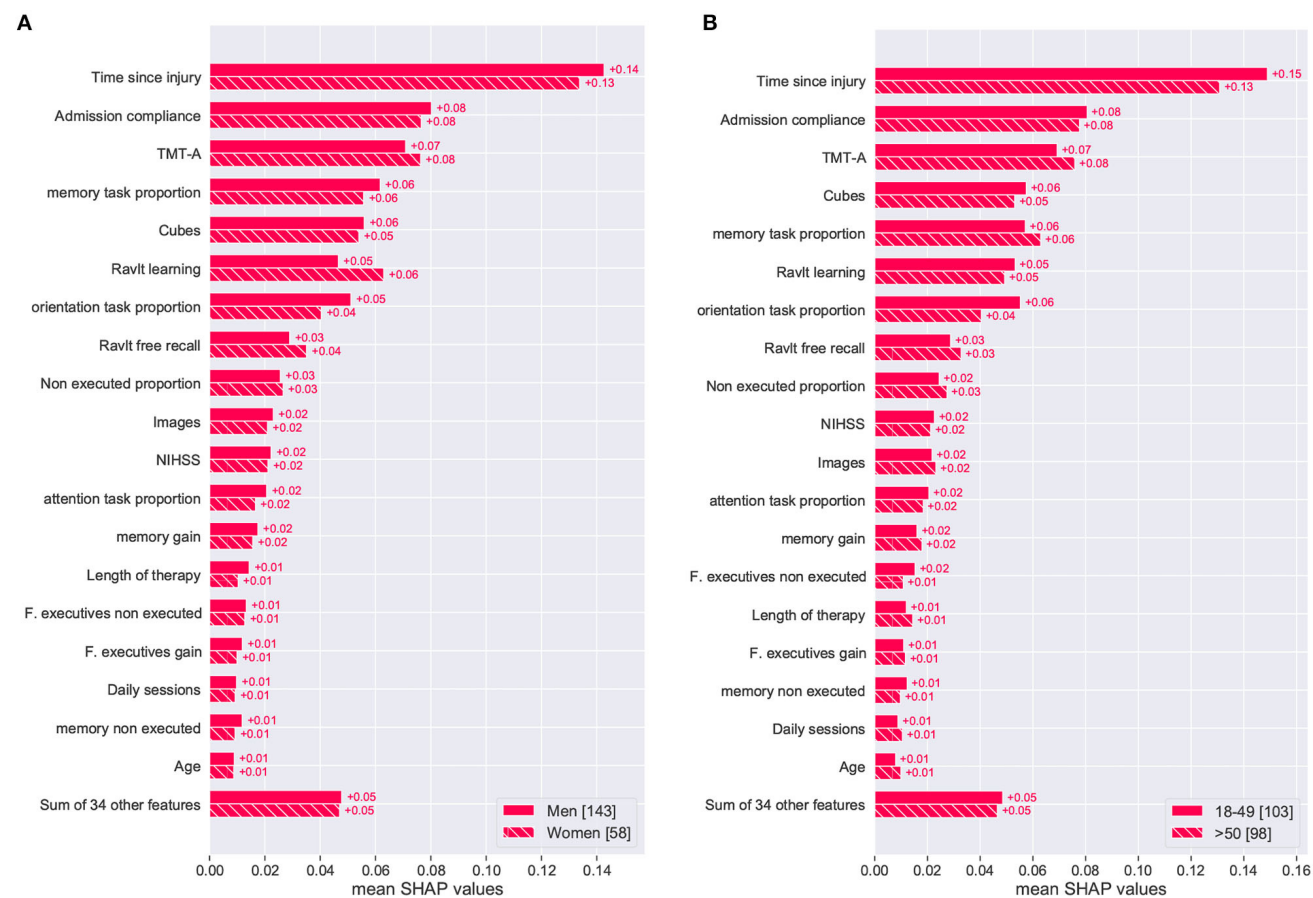
Global feature importance plot based on mean SHAP values for sex **(A)** and age **(B)** cohorts of patients. Results from the *XGBClassifier_All* optimized model.

Interpretation at a general level seeks to provide information for patient populations and the key parameters guiding the model's outcome. Through this type of analysis, relevant trends of outcomes and the features that influenced them can be identified. The SHAP method assigns different values to each feature based on their contribution to the model's outcome (positive or negative); these values are denoted as SHAP values. For the general interpretation analysis, the mean absolute SHAP values (for each feature) are computed over all entries in the dataset, and they are presented in Figure 6. The features were sorted based on their absolute impact on the model's outcome, and they are presented over two cohorts: sex (male and female) and age (below and above 50 years old). This visualization reveals if different features have a different effect depending on the sex of the patient or their age group.

Figures 6A,B show that the time since injury was the most determining feature for predicting the global improvement of a patient. This feature seemed to have a slightly stronger effect on men under 50 years of age. Admission compliance was identified as the second most important feature with no significant difference in terms of sex or age group. Out of the group of standardized cognitive assessments, TMT-A (attention), Cubes (visual), and Ravlt learning (memory) were among the most important features for the global improvement prediction. From this group, only Ravlt learning exhibits a noticeable difference within the sex cohort. As for therapy features, memory task proportion and orientation task proportion were the most important features for the model prediction. With respect to its effect across sex and age, orientation task proportion was the only variable showing a clear difference over these cohorts.

**Figure 7:**
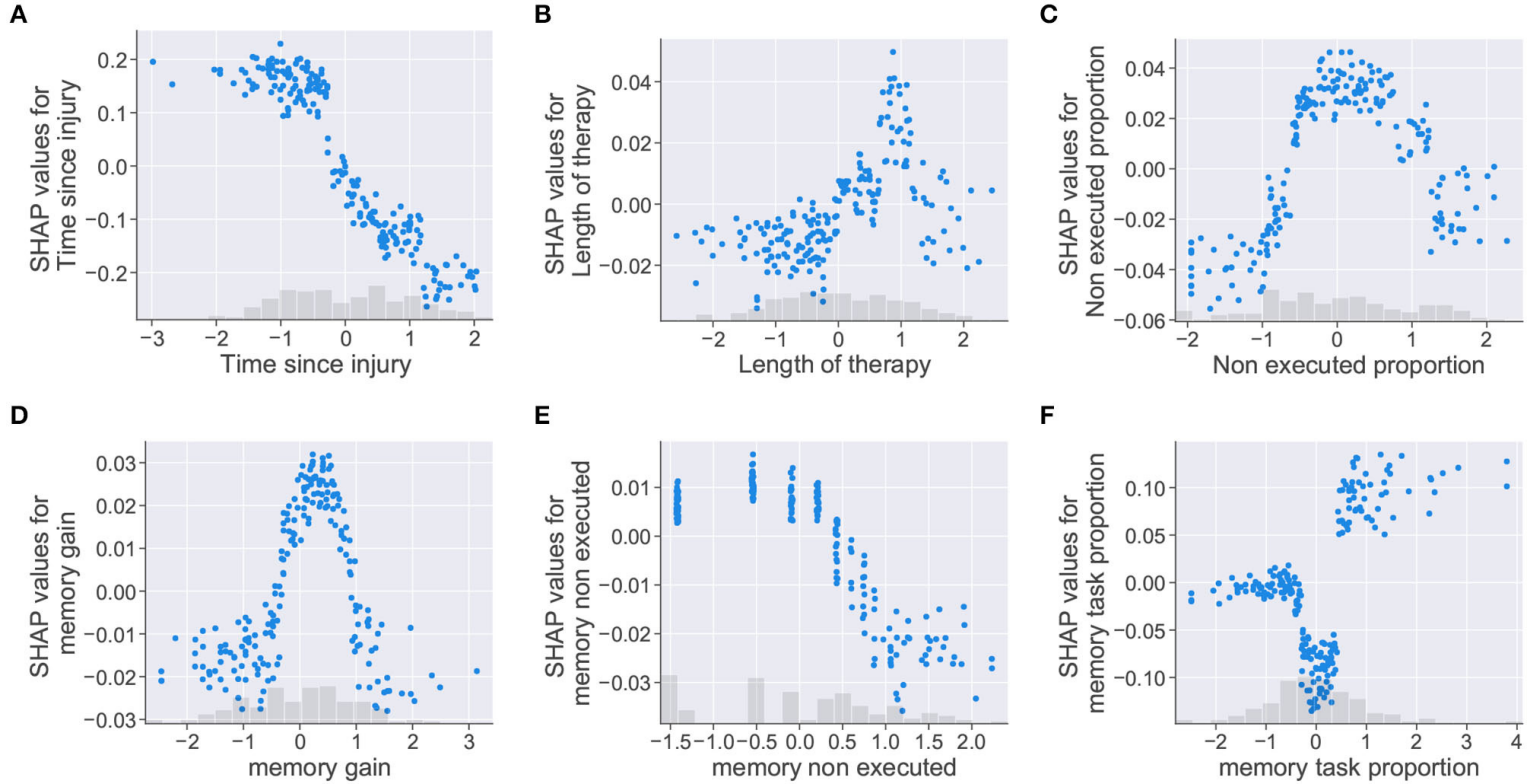
Dependence scatter plots showing the effect of six input features in the model predictions. SHAP values (y-axis) pushes the outcome toward a class “0” (no cognitive improvement, negative SHAP values) or a class “1” (cognitive improvement, positive SHAP values). Normalized instances of features with corresponding histograms depicted in the x-axis. **(A)** Time since injury, **(B)** Length of therapy, **(C)** Non executed proportion, **(D)** memory gain, **(E)** memory non executed, and **(F)** memory task proportion.

The isolated effect of features on the model's prediction was represented using the dependence scatter plots presented in Figure 7. These plots show the normalized range of values a feature can take vs. the SHAP value assigned to each instance in the dataset. The plots include a histogram at the bottom showing the distribution of the feature values. For this case study, negative SHAP values contribute to the patient being classified as class “0” (no cognitive improvement). Meanwhile, positive SHAP values push the model toward classifying the patient as class “1” (cognitive improvement). The dependence scatter plots presented in Figure 7 show how the trained model reacts to different feature instances and identifies what values the model considers relevant for the final decision.

Dependence scatter plots were generated for six input features, as observed in Figure 7. Scatter plots for the *time since injury, length of therapy*, and *non executed proportion* are presented in the first row; meanwhile, *memory gain, memory non executed*, and *memory task proportion* are presented in the second row. Being memory the cognitive domain with the highest proportion of assigned tasks (40.2%), the three features related to this cognitive domain were selected for analysis.

For the *time since injury*, the dependence plot in Figure 7A shows that the trained model considered that higher values for the feature corresponded to lower SHAP values. Therefore, the model interprets that delays in the start of the rehabilitation (high *time since injury* values) increase the chances of a patient being placed in the no improvement class (class “0”). For the *length of therapy* presented in Figure 7B, higher SHAP values are assigned to therapy lengths up to a certain point (approximately 1 on the normalized x-axis), after this point, SHAP values start decreasing. For this feature, it looks like the model identified an optimal length of therapy which pushes the decision toward a positive cognitive outcome (class “1”). The plot for *non executed proportion* in Figure 7C shows that higher SHAP values were given to feature instances falling in the [–1, 1] range on the x-axis. In this case, the model seemed to penalize instances where the proportion of non executed tasks was too low or too high.

Dependence plots generated for the memory features showed similarities with the previous cases. For *memory gain*, the plot presented in Figure 7D shows that the model penalized extreme feature instances as in Figure 7C. The model assumes that *memory gain* values (i.e., memory task executions falling in the optimal result range) increased the chances of a patient being classified as having cognitive improvement only at a defined range of values (between 0 and 1 in the normalized x-axis). The *memory non executed* feature, presented in Figure 7E, shows the same behavior as the *time since injury* feature. For this case, the model interprets that patients with low therapy compliance (i.e., large amounts of task executions with “0” results) are more likely to be classified as having no cognitive improvement. For *memory task proportion*, the dependence scatter plot showed three clusters corresponding to different ranges of the feature values. Although the model interprets that patients with high *memory task proportion* values have more chances of being classified as an improvement case; mid values seemed to push the patient toward a no improvement outcome, even more than small values for the same feature.

#### 4.4.2. Individual Interpretation

The AI model's predictions need to be presented to clinicians with enough information to contextualize and justify their outcomes. At an individual level, a report of feature importance allows clinicians to compare the rationale of the model and their own medical criteria, especially when they do not align together (53). The individual interpretation analysis was focused on specific patients and their particular conditions to observe the model's behavior at an individual level. For this analysis, four cases were selected to present the model's inner decision workings: two patients reporting the highest *global improvement* scores and two reporting the lowest scores.

**Figure 8:**
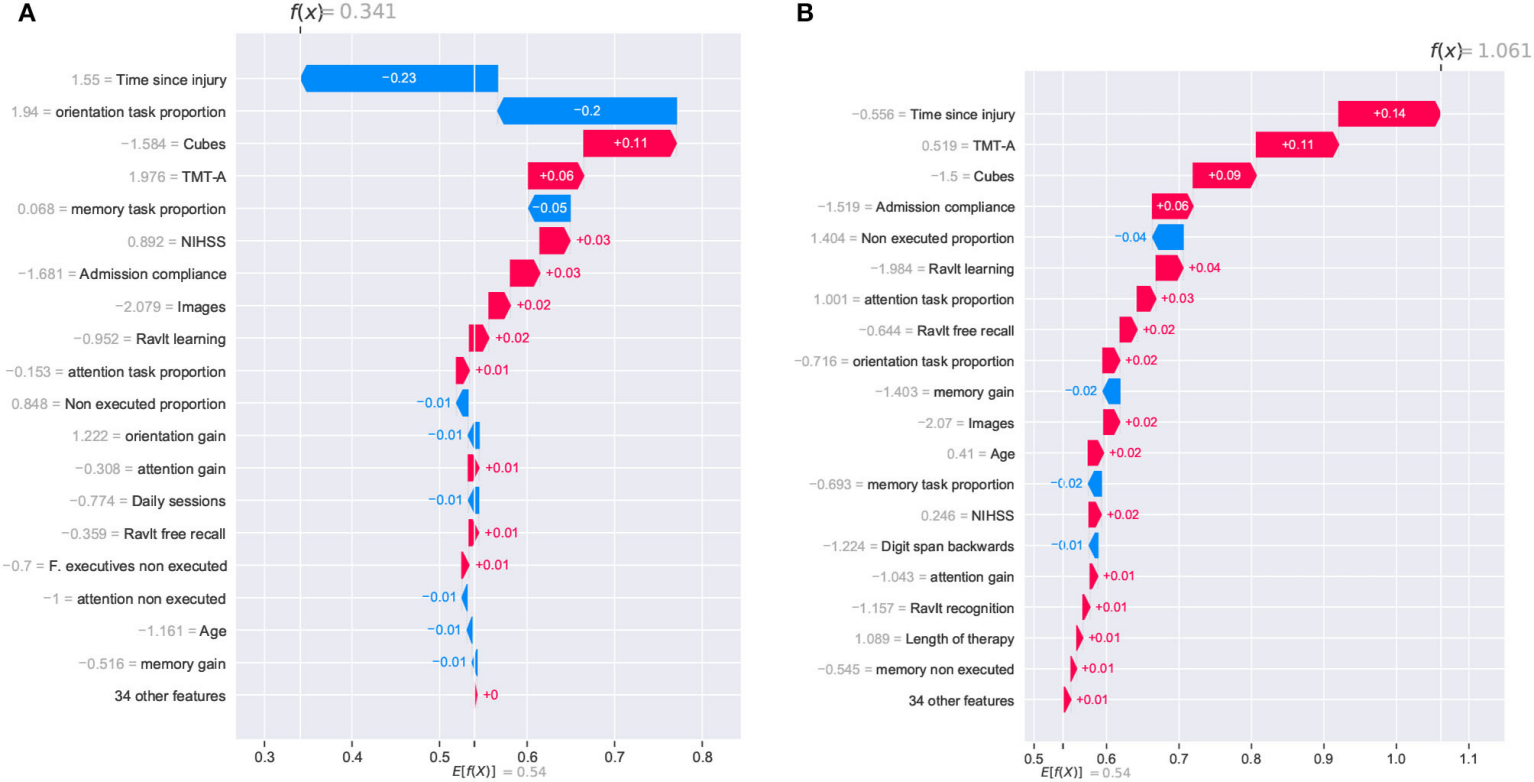
SHAP waterfall plots for explanations of individual predictions. Illustrative cases of two patients with the highest *global improvement* scores. Positive feature effects are represented in red and negative effects in blue. The plot should be read from *E*[*f*(*x*)], the expected value of the model output, toward *f*(*x*), the model output. Positive outcomes in the x-axis (in log-odds units) are probabilities above 0.5 of classifying a patient as a cognitive improvement case (class “1”). **(A)** Patient_123 and **(B)** Patient_133.

**Figure 9:**
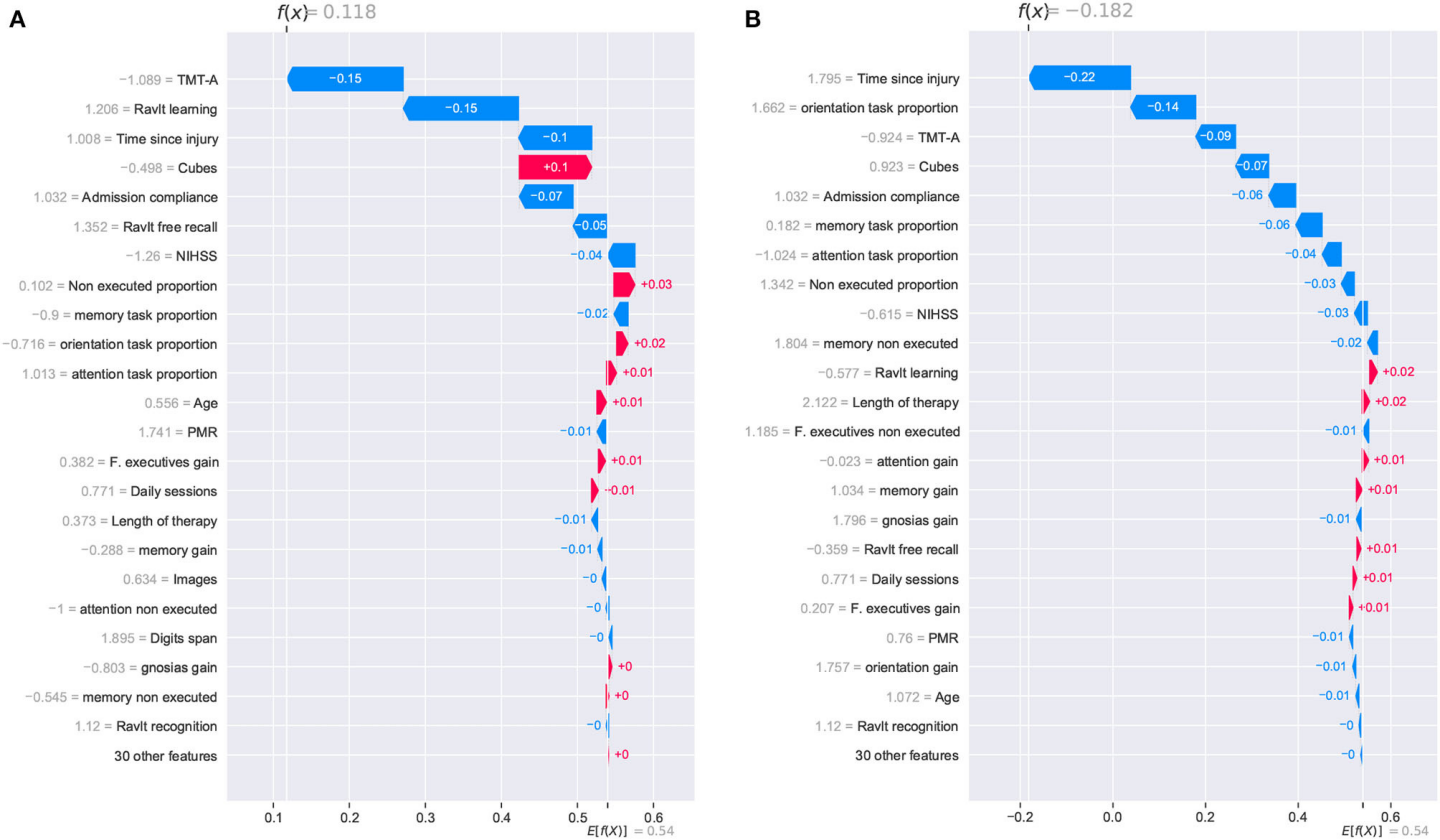
SHAP waterfall plots for explanations of individual predictions. Illustrative cases of two patients with the lowest *global improvement* scores. Positive feature effects are represented in red and negative effects in blue. Plot should be read from *E*[*f*(*x*)], expected value of the model output, toward *f*(*x*), the model output. Negative outcomes in the x-axis (in log-odds units) are probabilities above 0.5 of classifying a patient as a no cognitive improvement case (class “0”). **(A)** Patient_27 and **(B)** Patient_33.

The individual analysis was made using waterfall plots generated using the SHAP method; such plots are presented in Figures 8, 9. The plots show how each feature affects positively (red row) or negatively (blue row) the final decision of the trained model in each individual case. The plot should be read from the bottom to the top as it starts with the initially expected outcome (*E*[*f*(*x*)]) on the x-axis, which changes according to the effect of different features until reaching the final outcome (*f*(*x*)). SHAP reports the model's outcome in log-odds units (before the logistic link function). So, positive outcomes in the x-axis should be interpreted as probabilities above 0.5 of a patient having a positive cognitive improvement (class “1”).

The two patients with the highest *global improvement* scores were picked out and they were identified using their entry indexes in the dataset. These two patients, identified as *Patient_123* and *Patient_133*, were correctly classified by the *XGBClassifier_All* model as having a positive cognitive improvement (class “1”). The positive outcomes for *Patient_123* and *Patient_133* from the SHAP analysis (0.341 and 1.061, respectively) are presented in Figure 8. Although *Patient_123* was classified as a cognitive improvement case (positive outcome of 0.341), the waterfall plot shows that two features negatively affected the model's outcome (*time since injury* and *orientation task proportion*) pushing the model toward a no improvement prediction (class “0”).

Figure 8 shows that the *time since injury* had a different impact on both patients. This can be explained by observing the values corresponding to that feature. *Patient_133* reported a *time since injury* value of –0.556 (39 days in the original record) and an effect of +0.14; meanwhile, *Patient_123* reported a value of 1.55 (138 days in the original record) with an effect of –0.23. This analysis indicates that the model will lower the patient's chances of cognitive improvement if the *time since injury* is too high. Regarding the *orientation task proportion* effect, it is rather odd to observe a positive effect on the outcome of *Patient_133* (+0.02) considering that the original record shows a feature value of “0.” Based on this observation, it appears that the model lowered the chances of patients with specific amounts of orientation task executions. Finally, features like Cubes, TMT-A, and *admission compliance* positively affected both cases.

Two other patients reporting the lowest *global improvement* scores were selected for the individual analysis of the model. From this pair of patients, only one of them was correctly classified as a no improvement case (class “0”). For *Patient_27*, the SHAP analysis reported a positive outcome of 0.118, and it was erroneously considered an improvement case. Meanwhile, *Patient_33* reported a negative outcome of –0.182, and it was correctly classified as a no improvement case. Despite the erroneous classification of *Patient_27*, it is noticed that the reported outcome (0.118) was very close to reaching a negative value which would have resulted in the correct classification of the patient.

For these two cases, the prediction paths are presented in Figure 9. From the group of features that reported a strong contribution to the model's outcome, the cognitive assessment Cubes WAIS-III was the only one showing a completely inverse effect for both patients. As for the *time since injury*, although the effect was negative for both cases, the difference in the magnitude of the impact seemed to have been decisive for the model's outcome. For this feature, a difference in the start of the rehabilitation (105 days for *Patient_27* and 155 for *Patient_33*) resulted in almost 0.12, which is close to the 0.118 reported in the SHAP analysis. As observed in the previous cases, the model considers the *time since injury* as a determinant factor for the cognitive outcome of a patient.

## 5. Discussion

This study presented an ML model for cognitive improvement prediction of patients with ischemic stroke after therapy. To do so, several classifier algorithms were evaluated using varying input features from demographic, cognitive, and therapy health records. The study uses feature importance reports to analyze predictions from the best-performing model at general and individual levels. The analysis presented in this study helped to understand the underlying effect of different variables on the patients' cognitive outcomes. Moreover, it was possible to identify contributing therapeutic variables that resulted in a favorable outcome. Throughout the development process, the article brings attention to the role of data scientists and clinicians and the value of their input at each stage of the development process. In this section, reflections on each step of the process are presented, and a discussion is presented on how some of the findings of this study can be generalized and applied across other healthcare-related projects.

### 5.1. On the Data Exploration Process

The development process presented in this study begins with the data exploration phase. At this phase, the involvement of clinicians was key to building and discovering new variables, exploring the relationship among the feature set, identifying inconsistencies in the dataset, applying appropriate filters, and establishing strategies to deal with missing data entries.

In this phase, hand-crafted features derived from the therapy records were built using common aggregation functions like count, sum, or mean. Yet, other key variables that were not implicitly represented in these records were revealed only through the input of clinical experts. The analysis then showed that these features (e.g., *memory task proportion, orientation task proportion*) were strong indicators of the patient's cognitive outcome. In this study, we observed how a close interaction with domain experts leads to discovering important descriptive features. Although we relied on a specific platform (GNPT) to collect therapy information and build these descriptive features, the same approach can be adapted over similar computerized settings to derive similar features.

This study relied on correlation and clustering analyses to examine the relationship among the dataset features. Similar to what clinicians reported regarding model explainability (53), well-designed visualization tools are essential to facilitate the understanding and interpretation of data exploration findings. Using good visual reports was key to easing clinicians' interpretation of the findings. This article employed heatmaps and cluster scatter plots to expose relationships among the predictor variables and the target features. Evidence gathered from this analysis served to arrive at a collaborative decision on removing correlated variables to reduce the overlap in input variables (multicollinearity) or keeping them to mitigate data sparsity by complementing missing information.

Findings at this phase can be used to identify inconsistent entries and apply proper filters to assure the quality of the data. As advised in other studies, clinicians are encouraged to make sure that data from patients is valid and reliable, especially in a context where healthcare AI is gaining more relevance (58). Biased outcomes can be avoided if the proper filters are applied to the dataset before starting the modeling phase. This aspect is especially relevant when establishing procedures to treat missing data. This study relied on recommendations from both clinicians and data scientists to balance the size of the dataset and its descriptive capacity. Yet, several patients are still left out of the final training set due to their low number of cognitive assessments. As commented by neuropsychologists, there are several reasons why a patient completes few cognitive assessments at admission. In this study, the *admission compliance* feature tried to capture this information and use it to predict the patient's outcome. However, there could be patients with a particular condition for which neuropsychologists recommend a specific set of cognitive assessments. Capturing all clinical cases is a challenging task and requires a combined effort from clinicians and data scientists.

Similar to previous studies (53, 58), this article confirms the necessity of having a higher level of involvement from clinicians in the development process of ML tools. More specifically, stakeholders involved in the data collection process should be aware of the data quality requirements in the context of ML; thus, assuring less biased models due to data inconsistency.

### 5.2. On the Modeling Process

Following the data exploration, the modeling phase receives a curated dataset to train an ML model targeting an outcome variable from the dataset. Several methods, commonly applied in ML development projects, were used along with this phase. Data standardization and one-hot encoding for data pre-processing, ANOVA f-tests for feature selection, cross-validation for performance and accuracy estimation, and a grid-search for parameter optimization. The adoption of structured processes and validated methods guarantees an efficient development process and more reliable models (59, 60).

Compared to other development phases (data exploration and model explanation), clinical involvement might not be especially crucial during the modeling stage. Yet, as mentioned in several studies (53, 58, 61), healthcare personnel (e.g., clinicians, students, attending physicians) is encouraged to engage with ML key concepts, and standard methods as AI-based systems continue to gain relevance in the clinical domain. Better knowledge of the broad concepts in ML would increase the clinicians' trust and, ultimately, their adoption of the model. In addition, knowing how models function can help clinicians understand their limitations and, thus, guide their own expectations. This type of engagement seeks to bridge the collaboration between clinicians and data scientists, putting both on common ground.

### 5.3. On the Explanatory Analysis

Finally, the explanatory stage covered the tasks and techniques used to deliver the model's outcomes to clinicians. Model explainability is a critical field of research as it was understood that translating raw model predictions to clinical practice was not suitable since the poor justification of predictions affected the trust in the model and reduced the chances of it being adopted in a healthcare setting (53, 54). Clinicians identified feature importance reports as a desirable tool for rationalizing models' predictions and using them to compare their decision-making process (53). This study applied feature importance charts to explain the model's inner decision workings. The analysis was made at a general and an individual level which allowed us to observe the impact of different features over stratified patient populations and individual clinical cases.

From this analysis, it was interesting to observe how the model replicated some of the experts' reasoning regarding certain variables. For instance, for the *time since injury*, the results confirmed the importance of this feature and how larger values were determinant in predicting no improvement cases. Similarly, it was observed that extremely high values for the *non executed proportion* feature increased the probabilities of a patient being classified as a no improvement case. This behavior aligned with the comments from neuropsychologists in the sense that poor compliance during therapy was an indicator of a poor cognitive outcome. These results show that the trained model is, in fact, following similar decision criteria used by a clinician. Similarly, other features that have a critical role in the model's prediction should be analyzed to find a coherent behavior with the clinician's decision-making process. Although the model's prediction may not replicate the clinician's rationale, it can provide a perspective to support clinical decisions, even if it does by providing data supporting a counterargument or perspective.

The feature importance charts generated using the SHAP method served well to interpret the outcomes from the use case presented in this study. However, this approach might not apply to more complex ML methods. Depending on the context, alternative solutions like the local interpretable model-agnostic explanations (LIME), which target individual level explanations (62), and new explanation techniques for Deep Neural Network (DNN) models (63, 64) can be utilized.

### 5.4. On the Cognitive Improvement Prediction

The model presented in this article targeted the *global improvement* variable to predict the cognitive outcome after therapy. This variable was derived from the improvement indicators from admission and discharge cognitive assessments. As in other studies, this variable was adapted to fit a binary classification problem by establishing an improvement threshold: class “0,” *global improvement <=0*; class “1,” *global improvement>0*. Although this variable is not a standardized measure, it can easily be adapted to fit different stroke rehabilitation settings, where cognitive assessments are administered. The performance of the models was evaluated based on the Recall score to prioritize the correct identification of patients in the class “0.” The study showed the importance of informed decisions regarding the positive class, which directly affects the metric used for the learning and optimization of the model. As for AUC scores, we observed values above 0.5 for the initial evaluation of algorithms and after optimization. Similar to the other reported metrics, no significant improvement was noticed. These results showed that changing the algorithm or optimizing its parameters did not improve the model's performance. One alternative would be to deal with some of the data limitations, such as the class imbalance reported in Figure 5. Over-sampling techniques can be applied to observe how the model's performance responds. Another possible strategy would be adding more variables with higher discriminatory capacity (e.g., comorbidity indicators, additional standardized scales). This study has paid special attention to the data preparation phase. The techniques included in this phase are intended to facilitate the inclusion of new patients' information. It is expected that as new patients are included, adjustments can be made, and performance will improve. Alongside performance, the study seeks to set the foundation for a robust ML tool at different phases during the cognitive rehabilitation workflow. Moreover, future work will explore the application of oversampling techniques or data imputations to improve the performance of the model.

Very few studies have explored the use of therapeutic variables as predictor factors for cognitive outcomes after rehabilitation. The study presented in (22) included variables representing the load and type of rehabilitation activities (e.g., number of sessions, number of executed activities and mean number of executed activities per session) and used the Disability Ranking Scale (DRS) as the target variable. That study reflected on the importance of providing actionable information to therapists and how this can lead to the design of better intervention programs. Our study extended the granularity level of therapy variables by decomposing the therapy information across cognitive domains. This enables the delivery of detailed information on the specific cognitive domain impacting the overall cognitive outcome (e.g., *memory task proportion* and *orientation task proportion*). In addition, we introduce new variables that have shown promising results acting as predictor variables (e.g., *admission compliance*).

Although performance can be improved, possibly by including comorbidity or demographic information and using more sophisticated ML algorithms, the model showed its capacity to identify patients “at risk” (i.e., patients belonging to the class “0,” *global improvement <=0*). This information can be helpful to alert clinicians of cases where the therapy program requires adjustment. Using the trained model, outcomes for different therapy settings can be tested and reported using scatter charts to identify the configuration that maximizes the patient's cognitive improvement.

### 5.5. Limitations

The sparsity of the data was the main limitation of this study. As reported, features from the cognitive assessment records had very different completeness levels, forcing the removal of some variables to maintain an acceptable number of entries in the dataset. In terms of descriptive features, the study didn't consider some comorbidity indicators and some relevant demographic information like the level of education. For this study, *global improvement* was chosen as the model's target variable with a defined threshold used to frame the model output as a binary classification problem. Both the variable and the threshold used in this study still need further exploration to support their clinical significance. The threshold intends to separate patients with cognitive improvement from those that showed no improvement or got worse. Therefore, they must be considered just as referential indicators of a patient's cognitive status.

This study relied on specific cognitive assessments to act as cognitive outcome predictors; however, standardized scales for functional independence (e.g., FIM, BI) or degree of disability (e.g., mRS) in activities of daily living were not considered. Cognitive functioning has been reported to be strongly involved in the execution of activities of daily living. For example, Mori et al. (65) concluded independence in eating is strongly associated with cognitive improvement in subacute stroke. Functional independence has been extensively assessed in rehabilitation settings after stroke using standardized instruments such as the FIM or the Barthel index, which have not been considered in this study. This leaves room for future research, e.g., considering individual FIM items and their relationship to specific cognitive functioning assessments such as the TMT or RAVLT. Nevertheless, we have included stroke severity in our study, assessed using the NIHSS, the FIM has been reported in previous research to be strongly associated with the NIHSS. For example, according to Roth et al. (66) NIHSS correlated significantly with motor and cognitive FIM subscores for admission, discharge, and change measures.

### 5.6. Future Study and Direction

This study presented a model targeting the cognitive outcome prediction task. An extended model will be implemented adding more features to increase the accuracy of the model. Following a similar development methodology, other target features like *non executed proportion* will be considered to explore the level of therapy compliance of rehabilitation.

## 6. Conclusion

Stroke rehabilitation poses several challenges to clinicians, especially in building effective therapeutic programs that maximize patients' cognitive recovery. ML-based tools can assist clinicians in this task, but they need to produce actionable information and well-justified outcomes to increase their chances of adoption into a clinical setting. This article presented an ML prediction model that targets cognitive improvement after therapy for ischemic stroke surviving patients. Besides demographic and cognitive assessments variables, which are commonly used in literature, the model included therapeutic variables as predictor features. A large number of classifier algorithms were trained, optimized, and evaluated, varying the number of features received as input. A set of feature importance visual reports allowed the interpretation of the inner decision workings of the best-performing model. The study showed how certain therapeutic variables impacted the rehabilitation outcome for individual cases and across the entire study population. Potentially, clinicians can use this type of evidence to tune a therapeutic setting that maximizes the cognitive outcome.

## Data Availability Statement

The datasets generated for this study will be made available upon reasonable request to the corresponding author of the article.

## Ethics Statement

A specific written informed consent was not required for participants to be included in this study, in accordance with the local legislation and institutional requirements. Nevertheless at admission participants provide written informed consent to be included in research studies addressed by the Institut Guttmann hospital. The authors confirm that this study is compliant with the Helsinki Declaration of 1975, as revised in 2008 and it was approved by the Ethics Committee of Clinical Research of Institut Guttmann.

## Author Contributions

HM and AH conceived the study. HM, KC, and AG-R collected, selected, and cleaned the data. HM, KC, JK, AG-R, and AH statistically analyzed the data and received funding for this study. HM drafted the manuscript. JK, AG-R, KC, and AH revised the manuscript critically for important intellectual content and approved the final manuscript. All authors contributed to the article and approved the submitted version.

## Funding

This research was partially funded by PRECISE4Q Personalized Medicine by Predictive Modelling in Stroke for better Quality of Life-European Union's Horizon 2020 research and innovation program under grant agreement No. 777107, and by the Science Foundation Ireland (SFI) under Grant Number 17/RC/2289_P2.

## Conflict of Interest

The authors declare that the research was conducted in the absence of any commercial or financial relationships that could be construed as a potential conflict of interest.

## Publisher's Note

All claims expressed in this article are solely those of the authors and do not necessarily represent those of their affiliated organizations, or those of the publisher, the editors and the reviewers. Any product that may be evaluated in this article, or claim that may be made by its manufacturer, is not guaranteed or endorsed by the publisher.
